# Novel NAD^+^-Farnesal Dehydrogenase from *Polygonum minus* Leaves. Purification and Characterization of Enzyme in Juvenile Hormone III Biosynthetic Pathway in Plant

**DOI:** 10.1371/journal.pone.0161707

**Published:** 2016-08-25

**Authors:** Ahmad-Faris Seman-Kamarulzaman, Zeti-Azura Mohamed-Hussein, Chyan Leong Ng, Maizom Hassan

**Affiliations:** 1 Institute of Systems Biology, Universiti Kebangsaan Malaysia (UKM), 43600 UKM, Bangi, Selangor, Malaysia; 2 School of Biosciences and Biotechnology, Faculty of Science and Technology, Universiti Kebangsaan Malaysia, 43600 UKM, Bangi, Selangor, Malaysia; CNRS, FRANCE

## Abstract

Juvenile Hormone III is of great concern due to negative effects on major developmental and reproductive maturation in insect pests. Thus, the elucidation of enzymes involved JH III biosynthetic pathway has become increasing important in recent years. One of the enzymes in the JH III biosynthetic pathway that remains to be isolated and characterized is farnesal dehydrogenase, an enzyme responsible to catalyze the oxidation of farnesal into farnesoic acid. A novel NAD^+^-farnesal dehydrogenase of *Polygonum minus* was purified (315-fold) to apparent homogeneity in five chromatographic steps. The purification procedures included Gigacap S-Toyopearl 650M, Gigacap Q-Toyopearl 650M, and AF-Blue Toyopearl 650ML, followed by TSK Gel G3000SW chromatographies. The enzyme, with isoelectric point of 6.6 is a monomeric enzyme with a molecular mass of 70 kDa. The enzyme was relatively active at 40°C, but was rapidly inactivated above 45°C. The optimal temperature and pH of the enzyme were found to be 35°C and 9.5, respectively. The enzyme activity was inhibited by sulfhydryl agent, chelating agent, and metal ion. The enzyme was highly specific for farnesal and NAD^+^. Other terpene aldehydes such as *trans*- cinnamaldehyde, citral and *α*- methyl cinnamaldehyde were also oxidized but in lower activity. The *K*_m_ values for farnesal, citral, trans- cinnamaldehyde, α- methyl cinnamaldehyde and NAD^+^ were 0.13, 0.69, 0.86, 1.28 and 0.31 mM, respectively. The putative *P*. *minus* farnesal dehydrogenase that’s highly specific towards farnesal but not to aliphatic aldehydes substrates suggested that the enzyme is significantly different from other aldehyde dehydrogenases that have been reported. The MALDI-TOF/TOF-MS/MS spectrometry further identified two peptides that share similarity to those of previously reported aldehyde dehydrogenases. In conclusion, the *P*. *minus* farnesal dehydrogenase may represent a novel plant farnesal dehydrogenase that exhibits distinctive substrate specificity towards farnesal. Thus, it was suggested that this novel enzyme may be functioning specifically to oxidize farnesal in the later steps of JH III pathway. This report provides a basic understanding for recombinant production of this particular enzyme. Other strategies such as adding His-tag to the protein makes easy the purification of the protein which is completely different to the native protein. Complete sequence, structure and functional analysis of the enzyme will be important for developing insect-resistant crop plants by deployment of transgenic plant.

## Introduction

The challenges of insect pest control in the twenty-first century are the attempts to reduce the use of synthetic insecticides and use novel methods or biorational control approach for insect pest control [1]. Therefore, new strategy which is safer and practical nature, such as interfering the hormonal control of insect development and reproduction were proposed [2–4].

Juvenile hormone III (JH III) was originally known for its ability to maintain juvenile character of insect larvae and thus ensure the proper onset of metamorphosis [5]. The JH III was secreted by a pair of very tiny glands, the corpora allata, which form part of the whole brain complex of insect [6]. The hormone plays a major role in regulating both development and reproductive maturation in insects [7–9]. Sufficient JH III will promote larval to larval molt (juvenile stage), while low or absence of JH III will cause the larvae to undergo larval to pupal molt that initiate metamorphosis or nymphal adult transformation [1,10]. Supply of JH III makes an insect reiterate its juvenile stage, whereas removal of JH III causes the insects to metamorphose prematurely [11]. As exposure of JH III can easily deflect the insects developmental pathways, JH III was studied for agricultural insect pest control [12] as third generation pesticides [2] and spotlighted as safe targets for the eco-friendly insecticides [13–16].

The JH III biosynthetic pathway involves 13 discrete enzymatic steps organized in an obligatory sequence [17]. The early steps in the biosynthetic pathway of insect JH III involve the mevalonate pathway (MVAP) to form farnesyl pyrophosphate (FPP) [18]. FPP synthase (FPPS), a short-chain prenyltransferase is responsible to generate FPP by completing two sequential head-to-tail couplings involving a dimethylallyl pyrophosphate (DMAPP) and two isopentenyl pyrophosphate (IPP) [17]. In the later steps of JH III biosynthetic pathway, FPP phosphatase (FPPase) efficiently hydrolyzes FPP to farnesol [19]. Farnesol will then undergoes two sequential oxidation reactions catalyzed by one or two NAD^+^-dependent dehydrogenase(s) [20] that generate farnesal and farnesoic acid [17]. While the first reaction was catalysed by either a farnesol oxidase [21] or farnesol dehydrogenase [22,23], the enzyme catalysing the second reaction has yet to be isolated and characterized [1]. In orthopteran and dictyopteran insects, farnesoic acid is first methylated to methyl farnesoate, which in turn undergoes a C10, C11 epoxidation to JH III [24]. These steps were generally considered to be JH-specific, Nontheless the isolation, identification and biochemical characterization of these enzymes was hindered by the small size of the corpora allata gland [25].

On the other hand, JH III and its biosynthetic precursor in insects were also identified in plants including sedges, *Cyperus iria* L., *C*. *aromaticus* [26] and deciduous tree, *Cananga latifolia* [15]. Compared to insects, their presence in plants is poorly understood [27]. It was proposed to form part of a defensive strategy of the plant [15,28] which demonstrate a novel plant defence mechanism against insects herbivory [26]. From a biotechnological and food-developmental point of view, understanding and manipulating the defence systems of plants is of course of huge interest [29].

Lately, farnesol have been identified in the essential oils of *Polygonum* sp. [30,31]. Enzyme activities of farnesyl pyrophosphate synthase, farnesol dehydrogenase and farnesal dehydrogenase have also been detected in cell-free extracts of *Polygonum minus* [23] suggesting that JH III pathway might exist in *P*. *minus*.

*P*. *minus* is an annual herb found to possess a wide range of medicinal properties [32–35]. *P*. *minus* was classified in magnoliophyta division, polygonales order, polygonaceae family and genus of Polygonum [36]. To elucidate the JH III pathway in plant, we described the purification and characterization of NAD^+^-dependent farnesal dehydrogenase that responsible for the conversion of farnesal to farnesoic acid from *P*. *minus* leaves. This article is the first to reports the purification and characterization of farnesal dehydrogenase enzyme from plant. The characterization of this enzyme will provide valuable information on understanding the JH biosynthetic enzymes and their role in plants, which may help in developing insect-resistant crop plants. Developing crop plants which resistance to insect herbivores, would be a significant gain for the food and production industry, both at an economical and environmental level [29].

## Materials and Methods

### Plant materials and chemicals

The leaves of *P*. *minus* used for the study were obtained from plants growing in an experimental field of the Institute of Systems Biology, Universiti Kebangsaan Malaysia (UKM). Farnesal (mixture of isomers), *p*-cumic aldehyde, *S*-perillyl aldehyde, coniferyl aldehyde and vanillin were purchased from Sigma Aldrich (St. Louis, USA). Citral (mixture of geranial and neral), *trans*-cinnamaldehyde, α-methylcinnamaldehyde, citronellal, carvone, 4-dimethylaminocinnamaldehyde, α-amylcinnamaldehyde, veratraldehyde and salicylaldehyde were from Tokyo Chemical Industry (TCI) (Tokyo, Japan). α-Bromocinnamaldehyde, 5-bromovanillin, isovanillin, and ethylvanillin were obtained from Alfa Aesar (Ward Hill, MA). *Trans*, *trans*-farnesoic acid was from Echelon Biosciences Inc. (Salt Lake City, USA). Toyopearl GigaCap S-650M, Toyopearl GigaCap Q-650M, Toyopearl AF-Blue HC-650M, and TSK Gel G3000SW were from Tosoh Bioscience (Tokyo, Japan). Standard proteins for size exclusion chromatography were from Bio-Rad (Hercules, CA). The PageRuler^™^ Prestained Protein Ladder, ~10–170 kDa (SM0671) from Fermentas, USA was used as a standard protein marker for polyacrylamide gel electrophoresis sodium dodesyl sulphate (SDS-PAGE). All other reagents were commercial products of analytical grade. Water-insoluble chemicals were dissolved in ethyl acetate or dimethyl sulfoxide (DMSO), and subsequent dilutions were conducted in the water with final concentration of 0.34% (v/v). The presence of ethyl acetate or DMSO in the reaction mixture had no effect on enzyme activity.

### Preparation of cell-free extract

The extraction method was modified from the method used by Hassan et al (2012) [37]. Approximately 200 g (fresh wt.) of *P*. *minus* leaves were frozen in liquid nitrogen and ground to a fine powder with a Waring blender. The frozen powder was immediately slurried with cold extraction buffer (100 mM tricine-NaOH buffer (pH 7.5) containing 2.5 mM of 2-mercaptoethanol (2-ME), 25% (v/v) of sucrose, 5 mM of thiourea, 1 mM of phenylmethylsulfonylflouride, 1 mM EDTA, 50% (w/w) amberlite XAD-4, and 10% (w/v) polyvinylpolypyrrolidone for 15 min before being squeezed through four layers of cheesecloth. The crude homogenate was centrifuged at 20,000 × *g* at 4°C for 30 min to remove cell debris. The pellets, which were devoid of enzyme activity, were discarded and the supernatant, which contained farnesal dehydrogenase activity, was used as the enzyme source.

### Protein measurement

Protein concentration was measured by the Lowry method [38], using bovine serum albumin as the standard, or by absorbance at 280 nm. The proteins eluted from column chromatography were monitored by measuring absorbance at 280 nm.

### Enzyme assay

Farnesal dehydrogenase activity was measured by observing the increase in absorbance at 340 nm at 35°C [23]. The standard reaction mixture (1.5 mL) contained 100 mM of glycine-NaOH buffer (pH 9.5), 2.0 mM of farnesal in ethyl acetate, 1.0 mM of NAD^+^, and an appropriate amount of enzyme (6 μg). The reaction was started by the addition of cell-free extract or purified enzyme. Enzyme activity was calculated using an extinction coefficient of 6,200 M^-1^∙cm^-1^ for NADH. One unit of enzyme activity was defined as the amount of enzyme that catalyzed the formation of 1 μmol of NADH per min under the assay conditions [39]. Specific activity was defined as units of enzyme activity per mg protein.

### Purification of farnesal dehydrogenase

Purification of farnesal dehydrogenase was performed at 4°C. The flow rate for the column chromatographic steps was maintained at 1.3 mL/min. Throughout the purification procedure, 100 mM tricine-NaOH buffer (pH 7.5) containing 2.5 mM of 2-ME (buffer A), was used, unless otherwise specified.

The cell-free extract (6640 mg protein) was loaded onto a Toyopearl GigaCap S-650M column (1.6 × 70 cm) equilibrated with buffer A. The column was washed for four column volumes (7 mL/fraction) with buffer A and the protein was then eluted for four column volumes with the same buffer containing 2 M KCl.

Unbound proteins (133 mL) with farnesal dehydrogenase activity were collected and subjected to a Toyopearl GigaCap Q-650M column (1.6 × 70 cm) pre-equilibrated with buffer A. The column was washed with four column volumes (7 mL/fraction) of buffer A and the protein was then eluted for four column volumes using the same buffer supplemented with 2 M KCl.

Unbound proteins with enzyme activity (56 mL) were pooled and applied to a Toyopearl AF-Blue HC-650M (1.6 × 15 cm) column equilibrated with buffer A. The column was washed with four column volumes (5 mL/fraction) of buffer A and the protein was then eluted for four column volumes of the same buffer containing 1 M KCl.

The unbound fractions with enzyme activity (40 mL) were then pooled and concentrated with a Vivaspin^®^ 2 sample concentrator (molecular weight cut-off, 3 000) (GE Healthcare Bio-Sciences AB, Uppsala, Sweden) and loaded for two times onto (0.75 cm × 30 cm) TSK Gel G3000SW equilibrated with buffer A. Elution was performed at a flow rate of 0.5 mL/min and 0.5 mL/fractions were collected.

Finally, fractions containing enzyme activity were pooled and stored at -80°C until further use. The proteins eluted from column chromatography were monitored by measuring absorbance at 280 nm. The purity of the enzyme was determined by polyacrylamide gel electrophoresis (SDS-PAGE).

### Product identification of farnesal dehydrogenase by gas chromatography-mass spectrometry (GC-MS)

Identification of reaction product was performed using gas chromatography-mass spectrometry (GC-MS) analysis according to the method described by Shinoda and Itoyama [40] with slight modifications. Farnesal (2.0 mM) was incubated in 100 mM of glycine-NaOH buffer (pH 9.5) containing 1.0 mM of NAD^+^ with purified farnesal dehydrogenase (24 μg). Negative control was done by using the same mixture as mentioned above without addition of enzyme. After a 3 hour of incubation at 37°C, 500 μl of ethyl acetate was added, and the mixture was briefly vortexed. The ethyl acetate layer was immediately subjected to GC-MS analysis at Laboratory of Molecular Structure Characterization, Centre for Research and Instrumentation Management (CRIM), UKM. The GC-MS analysis was performed on an Agilent 7890A gas chromatograph (GC) directly coupled to the mass spectrometer system (MS) of an Agilent 5975C inert MSD with triple-axis detector equipped with a capillary DB-5MS UI column (30 m, 0.25 mm, film thickness of 0.25 μm). The sample (1 μl) was injected with an Agilent G4513a ALS (Automatic Liquid Sampler). The temperature of the column was set initially at 40°C for 3 min and then increased by 5°C per min to a final temperature of 220°C. Helium was used as carrier gas at flow rate of 1 mL per min. Data acquisition and processing were performed using MSD Chemstation software. Compounds separated on the column were identified by comparing their retention time and mass fragmentation patterns with authentic standards and library matches.

### Measurement of molecular mass and isoelectric point

The native molecular mass of the enzyme was estimated by size exclusion chromatography on a TSK Gel G3000SW column (0.75 cm × 30 cm) equilibrated with 0.1 M Tris-HCl buffer (pH 7.5) containing 2.5 mM of 2-ME. The purified enzyme (73 μg) was dialyzed at 4°C against 1.5-liter of 100 mM Tris-HCl buffer (pH 7.5) for overnight with three changes. The standard proteins used were γ-globulin, ovalbumin, myoglobin, and vitamin B_12_.

SDS polyacrylamide gel electrophoresis (SDS-PAGE) was performed using a 12.5% polyacrylamide gel using the Laemmli method [41]. The PageRuler^™^ Prestained Protein Ladder, which has a standard-protein molecular-weight range of approximately 10–170 kDa (Product SM0671, Fermentas, St. Leon-Rot, Germany), was used as molecular marker. The gel was stained with silver staining using PlusOne Silver Staining Kit, Protein (GE Healthcare, Uppsala, Germany) according to the manufacturer’s instructions.

Isoelectric focusing was performed with an 18 cm ReadyStrip IPG strip (pH 3–10) (GE Healthcare Bioscience, Uppsala, Sweden). The strip was passively rehydrated with 0.2 μg of purified farnesal dehydrogenase in rehydration buffer (8.0 M urea, 4% (w/v) CHAPS, 0.5% (v/v) of pH 3–10 ampholites, 30 mM 2-ME, and 0.002% bromophenol blue) for 12 h. The isoelectric focusing was carried out using Ethan IPGphor (GE Healthcare Bioscience, Uppsala, Sweden) according to the manufacturer’s instructions. The strip was silver stained.

### Protein identification by MALDI-TOF/TOF mass spectrometry (MALDI-TOF/TOF-MS/MS)

Identification and analysis of the purified protein were carried out by peptide mass fingerprinting using MALDI-TOF/TOF mass spectrometry (MALDI-TOF/TOF-MS/MS). The purified farnesal dehydrogenase (0.6 mg) was dialyzed against 100 mM Tris-HCl buffer (pH 7.5) overnight with two changes before the protein solution was sent to the Proteomics Facility, Medical Biotechnology Laboratory, Faculty of Medicine, University of Malaya, Kuala Lumpur, Malaysia for mass spectrometry analysis. In-solution trypsin digestion, protein extraction and mass spectrometry analysis by MALDI-TOF/TOF-MS/MS were carried out according to the protocols described by [42] with slight modification. The mass spectrum was processed using Global Protein Server Explorer Version 3.6 software (Applied Biosystems). The peptide mass profiles were analysed using the Mascot search engine (Matrix Science, USA, http://www.matrixscience.com) to match the MS and MS/MS data against information in the database. The data were matched towards databases from mammalian, bacteria and plant downloaded from Swiss-Prot/TrEMBL (http://www.expasy.ch/sprot). The peptide sequences were also blasted against NCBI non-redundant (nr) database (http://blast.ncbi.nlm.nih.gov/Blast.cgi). The following combined parameters were used in NCBI searches: Viridiplantae was set as the organism, and the search was applied to other known full-length sequences of terpene aldehyde dehydrogenases and aldehyde dehydrogenases from *Streptomyces afghaniensis* (geranial dehydrogenase; WP_020275925.1), *A*. *aegypti* (aldehyde dehydrogenase 3–1; AGI96738.1), *Artemisia annua* (aldehyde dehydrogenase 1; ACR61719.1), *Nandina domestica* (short chain dehydrogenase; ACN87275.1), *Microbacterium trichothecenolyticum* (geranial dehydrogenase; KJL42303.1), *Burkholderia vietnamiensis G4* (vanillin dehydrogenase; ABO56825.1), *Altererythrobacter atlanticus* (geranial dehydrogenase; AKH43916.1), *Pseudomonas citronellolis* (probable short-chain dehydrogenase; ABC69246.1), *Castellaniella defragrans* (geranial dehydrogenase; WP_043683932.1) and *Pseudomonas putida* (*p*-cumic aldehyde dehydrogenase; AAB62298.1).

### Effects of pH and temperature on farnesal dehydrogenase activity

The residual activity of farnesal dehydrogenase was measured after heat treatment at various temperatures of 25°C to 70°C for 10 min in 100 mM tricine-NaOH buffer (pH 7.5) containing 2.5 mM 2-ME. Residual activity was calculated as percent of the original activity in the unheated preparation.

The influence of pH on *P*. *minus* farnesal dehydrogenase activity was estimated by monitoring the enzyme activity between pH 4.5–10.5 using various buffers at a concentration of 100 mM. The following buffers were used: citrate buffers (pH 4.5 –pH 6.0), potassium phosphate buffers (pH 6.0 –pH 8.0), tris- HCl buffers (pH 8.0 –pH 9.0) and glycine- NaOH buffers (pH 9.0 –pH 10.5). The enzyme activity was defined as the percentage of the maximum activity level.

### Effect of inhibitors and metal ions on activity of enzyme

The effects of inhibitors and metal ions on the enzyme activity were examined. The activity of the enzyme was measured after incubation of purified farnesal dehydrogenase (2.7 μg) with various reagents and metal ions for 10 min at 35°C. The effect of inhibitors tested include *p*-chloromercuribenzoate, iodoacetamide, sodium azide, 1, 10-phenanthroline, and the metal ions tested include Ag^+^, Li^+^, Ca^+^, Cu^2+^, and Fe^3+^. Concentration for each of these inhibitors and metal ions was set to be 0.1 mM and the incubated enzyme was assayed according to the standard assay method as described before. These inhibitors and metal ions were widely used in many previous reports of aldehyde dehydrogenase [43–48], and thus were selected for this study. The enzyme activity obtained from the reaction mixture without any extra ion or inhibitor was taken as a control, corresponding to 100% relative activity.

### Substrate specificity and determination of kinetic parameter

**Fig 1:**
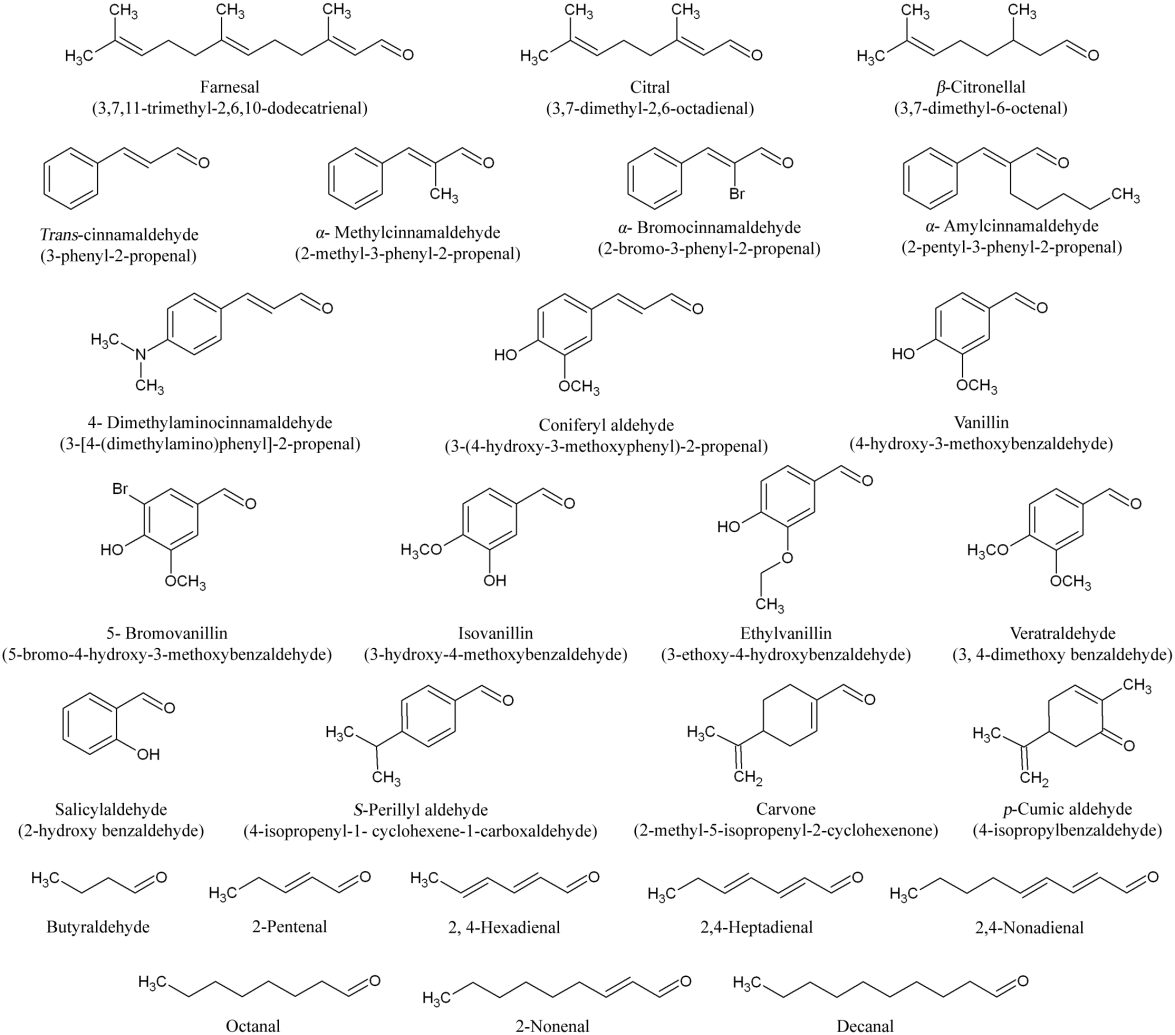
Aldehydes substrates used for the substrate specificity of farnesal dehydrogenase from *P*. *minus* leaves.

Activities of farnesal dehydrogenase on other substrates were measured as described in enzyme assay section. The standard reaction mixture contained glycine-NaOH buffer (100 mM, pH 9.5), substrate (2 mM), NAD^+^ (1 mM), and pure farnesal dehydrogenase (2.7 μg). There are 26 different aldehydes substrates tested in this study, namely citral, citronellal, *trans*-cinnamaldehyde, *α*-methylcinnamaldehyde, *α*-bromocinnamaldehyde, *α*-amylcinnamaldehyde, 4-dimethylaminocinnamaldehyde, coniferyl aldehyde, vanillin, isovanillin, 5-bromovanillin, ethylvanillin, veratraldehyde, salicylaldehyde, *p*-cumic aldehyde, *S*-perillyl aldehyde, carvone, decanal, 2,4-nonadienal, octanal, 2-nonenal, 2,4-heptadienal, 2,4-hexadienal, 2-pentenal and butyraldehyde (Fig 1). The effect of different substrate concentration, ranging from 0.1 mM to 2.5 mM with 0.5 increments on enzyme activity was estimated under optimal assay conditions (35°C, pH 9.5 and 5 min). The relative rate of oxidation for each substrate was determined as the percent of the enzyme activity measured with farnesal which was considered to correspond to 100%. The kinetic parameters, Michaelis-Menten constant (*K*_m_) and maximal reaction velocity (*V*_max_) were determined by linear regression from double-reciprocal plots according to Lineweaver- Burk [49]. The *K*_m_ and *V*_max_ were expressed in mM and μmol/min^−1^, respectively.

### Statistical analysis

All the characterizations of farnesal dehydrogenase from *P*. *minus* were assayed in triplicates. The data collected were presented as means ± standard deviation and also relative activity in percentage. The statistical analysis was performed using SPSS 18.0 software (SPSS Inc., Chicago, IL, USA).

## Results and Discussion

### Purification of farnesal dehydrogenase from *P*. *minus* leaves

JH III is biosynthesized in insect through catalyzation of several enzymes. Firstly, farnesol will undergo two steps of oxidation, which catalyzes by one or two NAD^+^-dependent dehydrogenase(s) to form farnesal and then farnesoic acid [20]. The oxidation of farnesal to farnesoic acid is still one of the less understood steps in JH III synthesis [14], despite farnesal oxidation has been predicted to be catalyzed by an aldehyde dehydrogenase. The aldehyde dehydrogenase was first revealed in *Drosophila melanogaster* using an *in vitro* colorimetric assay [50]. Moreover, there is also a report suggested that the oxidation of farnesal to farnesoic acid from adult female sphinx moth, *Manduca sexta*, was catalysed by NAD^+^-alcohol dehydrogenase which involved in the JH III biosynthetic pathway [20]. Recently, an NAD^+^-dependent class 3 fatty aldehyde dehydrogenase from *Aedes aegypti* was reported able to oxidize farnesal into farnesoic acid [14]. To date, all the enzymes of the JH-specific branch (later step) have been cloned and characterized [1,17–19,24] except for farnesal dehydrogenase, which yet to be isolated and characterized from any organism except from *A*. *aegypti* [1,7,14].

In this study, NAD^+^-farnesal dehydrogenase from *P*. *minus* was purified to apparent homogeneity with 312 fold purification and 2.5% recovery using ion-exchange column chromatography on strong cation exchange, Toyopearl Gigacap S-650M and strong anion exchange, Toyopearl Gigacap Q-650M, affinity column chromatography on Toyopearl AF-Blue HC-650M and size exclusion chromatography through Toyopearl TSK Gel G3000SW (Table 1). Activity of farnesal dehydrogenase was detected in unadsorbed fractions to both of the ion-exchange chromatographies.

**Table 1:** Purification summary of farnesal dehydrogenase from *P*. *minus* leaves.

Purification steps	Total activity (U)	Total protein (mg)	Specific activity (U/mg)	Purification (fold)	Recovery (%)
Cell -free extract	20.2	6640.0	3.0 × 10^−3^	1	100
Gigacap S 650M Toyopearl	17.9	638.6	2.8 × 10^−2^	9	89
Gigacap Q 650M Toyopearl	10.4	211.3	4.9 × 10^−2^	16	52
AF Blue 650ML Toyopearl	1.0	13.5	7.6 × 10^−2^	25	5
1^st^ TSK Gel G3000SW	0.6	0.6	9.4 × 10^−1^	310	3
2^nd^ TSK Gel G3000SW	0.5	0.5	9.6 × 10^−1^	315	2

The enzyme also did not bind to Toyopearl AF-Blue HC-650M column, which commonly used for the affinity isolation of dehydrogenases in spite of its NAD^+^-dependent dehydrogenase activity. Even though the enzyme did not bind to the resin for all the purification steps used, nevertheless, it is necessary for the purification of the *P*. *minus* farnesal dehydrogenase as these steps separate out most of contaminant proteins from the enzyme. The used of strong ion exchanger (Toyopearl GigaCap S-650M) instead of weak ion exchanger (Toyopearl DEAE-650M) may give high separation between the enzyme and other non targeted proteins. Elimination of these steps or changes in the type of the resin used, may result in higher impurities of the enzyme samples. The used of weak ion exchangers column chromatographic procedure in the preliminary experiment increased the specific activity to only 7-folds (data not shown), while strong ion exchangers column increased the specific activity up to 16-folds. After passage through the Toyopearl AF-Blue HC-650M column, the specific activity was further increased to approximately 25 times, with an overall recovery of approximately 5% of the initial activity (Table 1).

**Fig 2:**
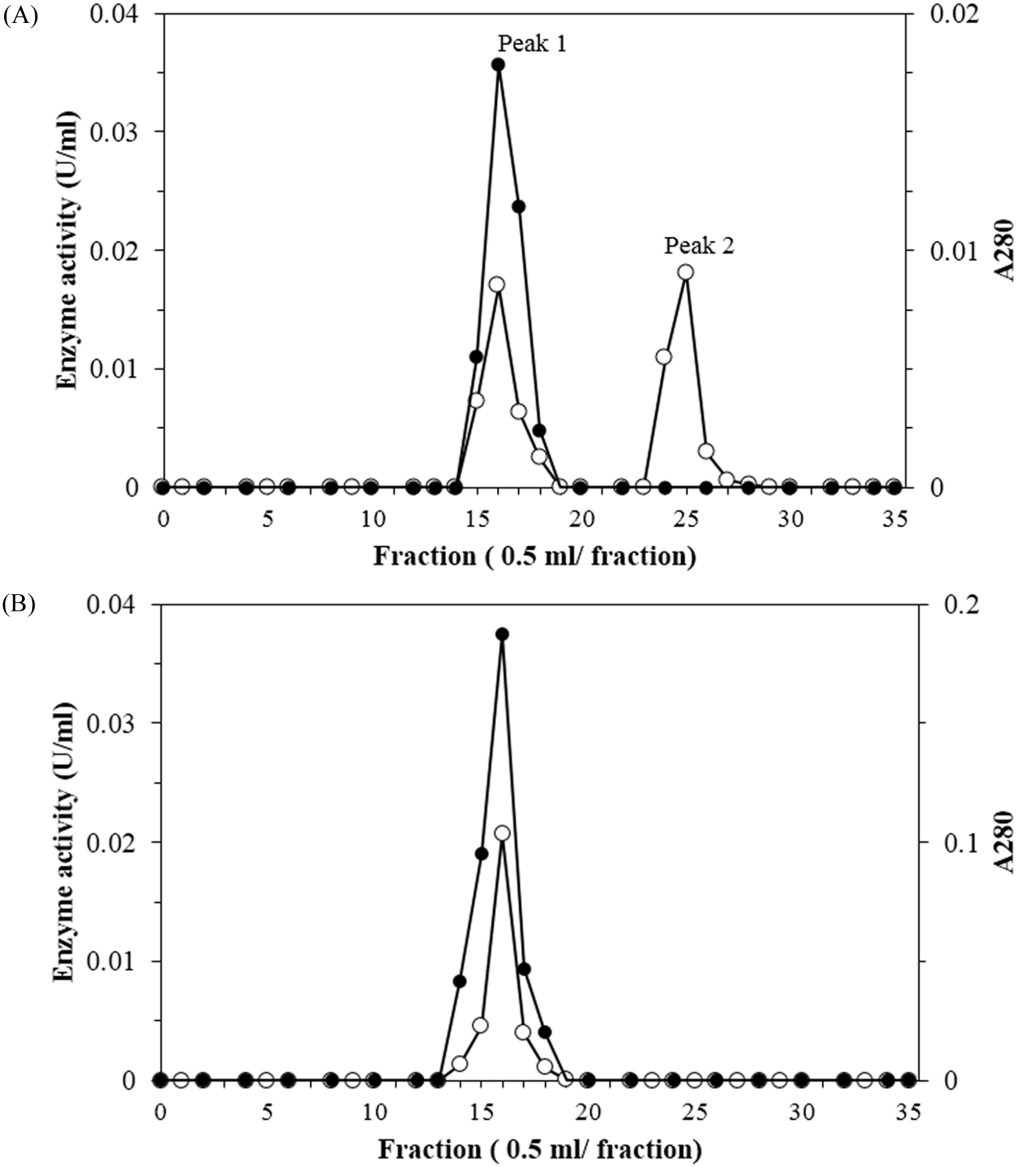
Elution pattern of activity and protein of *P*. *minus* farnesal dehydrogenase from size exclusion column chromatography (Toyopearl TSK Gel G3000SW) for first time (A) and second time (B). (●) Enzyme activity and (**○**) absorbance at 280 nm.

Farnesal dehydrogenase obtained was further purified using size exclusion column chromatography (TSK Gel G3000SW) column. This purification step was repeated twice. The elution profile was shown in Fig 2. The result shows that farnesal dehydrogenase activity was detected in peak 1 of Fig 2A. Analysis of fractions in peak 1 using Native-PAGE (S1A Fig) shows that there was only one protein which had closely similar mobility to the farnesal dehydrogenase. Therefore, peak 1 was further purified using the same column. The second purification step of size exclusion chromatography may serve to increase the length of the column where a longer column will give better separation and resolution of protein peak. Two consecutive steps of TSK gel G3000SW were successfully used to eliminate the non targeted protein as it was shown by a single protein band in Native-PAGE of S1B Fig. Hence, two successive steps of TSK gel G3000SW were crucial for purification of farnesal dehydrogenase to homogeneity. Farnesal dehydrogenase showed a 315-fold in purity and protein yields of 2%, after purification. The low yield was unavoidable at this stage as elimination of any steps or changes in type of the resin used during the purification may increase the impurities of the enzyme samples. The purity of farnesal dehydrogenase was further analysed using SDS-PAGE. The result which discussed under the determination of molecular weight and pI section shows that the SEC purified enzyme gave one single protein band while native-PAGE exhibited single band (S1B Fig) indicating high purity of the enzyme. Activity staining revealed the same position of the protein band capable of using farnesal as substrate suggested that the band belongs to farnesal dehydrogenase (S1B Fig).

### Product confirmation by GC-MS

**Fig 3:**
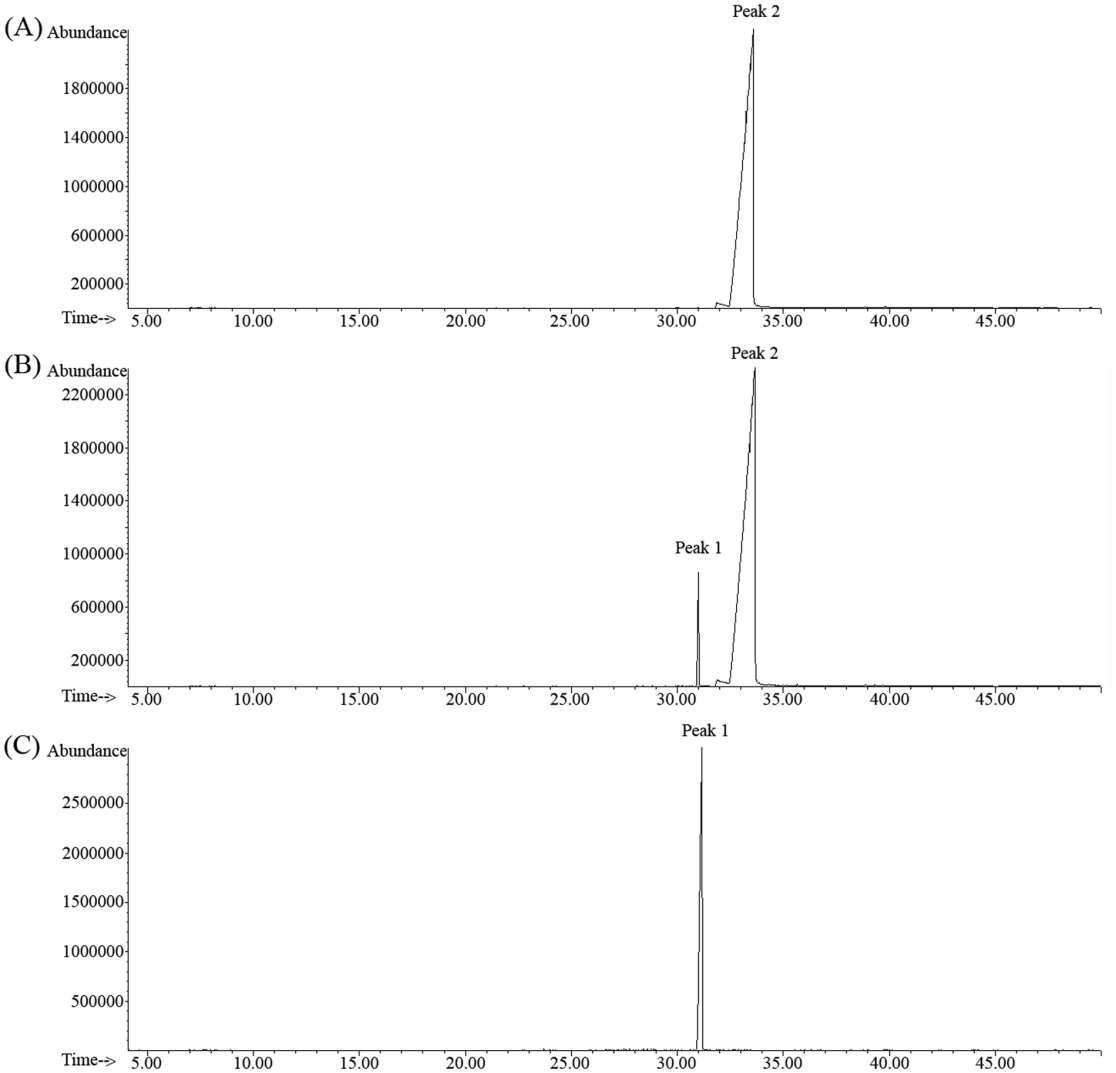
GC—MS analysis shows the purified *P*. *minus* farnesal dehydrogenase catalyse the farnesal into farnesoic acid. (A) Separation of authentic farnesoic acid. The enzyme assay reaction containing subsrate farnesal, coenzyme NAD^+^ and with (B) and without (C) purified farnesal dehydrogenase. The Peak 2 indicates the retention time of 33.7 min for authentic farnesoic acid (A) and enzymatically produced farnesoic acid (B). The Peak 1 indicates the retention time of 30.9 min for the substrate farnesal.

**Fig 4:**
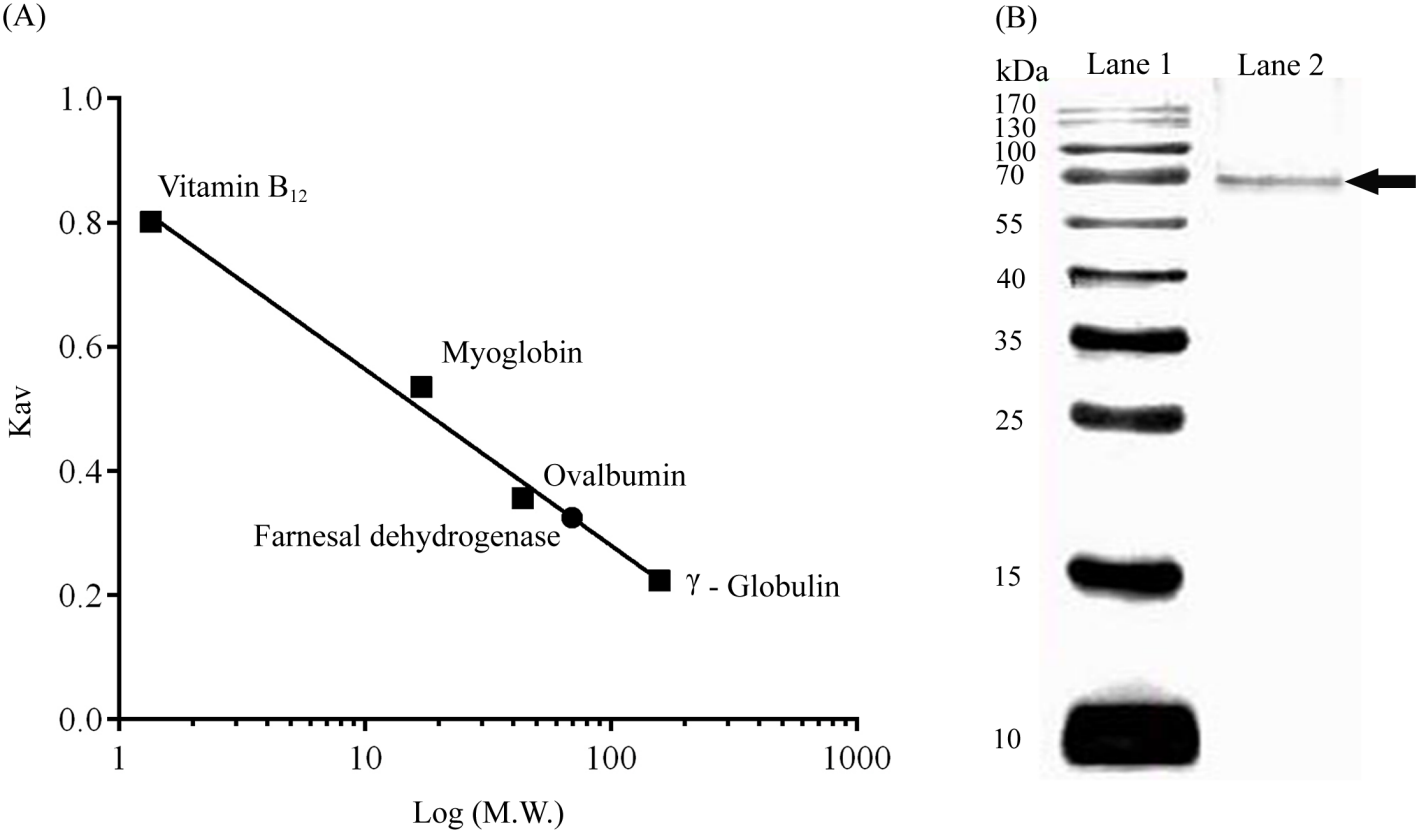
Calibration curve on TSK-gel G3000SW and molecular mass of the farnesal dehydrogenase from *P*. *minus*. (A) (●) Farnesal dehydrogenase (73 μg), (■) standard protein marker vitamin B_12_ (1.35 kDa), myoglobin (17 kDa), ovalbumin (44 kDa) and γ-globulin (158 kDa). The elution pattern of the protein size markers was linear on a semilog plot. Elution data are represented as log molecular weight to Kav. Kav was calculated as in the equation (Ve-Vo)/(Vt-V0), Ve, Elution volume; Vo, Void volume; Vt, total column volume. (B) Purified enzyme and standard proteins were subjected to electrophoresis in the presence of SDS with 12.5% gel. Lane 1, molecular weight marker. Lane 2, purified farnesal dehydrogenase (6 μg). The arrow indicates the protein band shown on SDS-PAGE.

To identify enzymatic product of the farnesal dehydrogenase reaction, GC-MS analysis was performed. An authentic farnesoic acid that served as positive control was first shown to have retention time of 33.7 min (Fig 3A). The enzyme reaction assay containing purified *P*. *minus* farnesal dehydrogenase (same sample as shown in Fig 4B), NAD^+^ and farnesal was incubated at 35°C for 3 hours before subjected for GC-MS analysis. Similar reaction was also prepared in the absence of the purified enzyme, to serve as negative control. As shown in (Fig 3B), a peak with a retention time of 33.7 min that similar to the retention time of authentic farnesoic acid was found in the reaction mixture that contains purified enzyme. This peak was not detected in the negative control reaction (Fig 3C). The GC-MS results confirmed that farnesal dehdyrogenase from *P*. *minus* was indeed catalyzing the oxidation reaction of farnesal into farnesoic acid. This result suggested that the purified enzyme is a farnesal dehydrogenase. For the first time, farnesal dehydrogenase was purified to homogeneity in plant.

SDS-PAGE (Fig 4B) shows that the SEC purified enzyme gave one single protein band and GC-MS analysis (Fig 3B) of the same fraction shows identification of farnesoic acid as the product. These result suggested that the purified enzyme was farnesal dehydrogenase.

### Determination of molecular weight and pI

The native molecular weight of farnesal dehydrogenase was estimated at ~70 kDa by size exclusion chromatography on the TSK Gel G3000SW column (Fig 4A). The subunit composition of the purified farnesal dehydrogenase was analyzed by SDS-PAGE under reducing conditions. The purified enzyme gave a single band of approximately 70 kDa on electrophoresis in the presence of SDS (Fig 4B). In agreement with the size exclusion chromatography result, we propose that the native form of farnesal dehydrogenase is likely a ~70 kDa monomeric protein in solution.

The molecular size was comparable with plant aldehyde dehydrogenase from *Oryza sativa* which have molecular weights of 66.2 kDa [51] while aldehyde dehydrogenase from *Arabidopsis thaliana* and *Ipomoea batatas* shows a bigger size of 112 kDa and 150 kDa, respectively [52,53]. In contrast, aldehyde dehydrogenases with molecular weight ranging from 50 kDa to 212 kDa, appeared in dimeric or tetrameric form also found in other organism [54–58].

The pI of *P*. *minus* farnesal dehydrogenase enzyme was determined by IEF as 6.6 (S2 Fig), similar to the pI of aldehyde dehydrogenase from *P*. *chrysosporium* (6.1) [59] and *H*. *sapiens* (6.3) [60]. Many other aldehyde dehydrogenase enzymes from various sources such as *Halobacterium salinarum*, *Rhodococcus erythropolis*, *Phanerochaete chrysosporium*, *Homo sapiens*, and *A*. *aegypti* have shown a broad range of isoelectric points (4.7–7.9) [14,45,59–61].

Referring to the result of protein purification, the enzyme was collected in the unbound fraction regardless of resin types used. The result suggests that the narrow gap between the pI (6.6) and the pH (7.5) of the buffer used in purification explained why farnesal dehydrogenase was recovered in the flow through fractions. Farnesal dehydrogenase was likely to have no net charge or weakly charged which makes no strong interaction with a charged medium [62]. Nevertheless, we do not rule out that other factors besides the net charge, the isoelectric region of a protein, surface charge distribution, protein hydrophobicity, van der Waals interactions, and choice of adsorbent materials can also influence the binding behaviour of proteins to the resin used in purification steps [63]. Note that, increasing or decreasing of pH in buffer which will help the bound of enzyme to the matrix could not be manipulated as it will affect enzyme activity and stability. The enzyme was very sensitive towards pH changes and would lose its activity when the buffer’s pH was adjusted.

### Analysis of the protein sequence by MALDI-TOF/TOF-MS/MS

Mass spectrum data of MALDI TOF/TOF-MS/MS for farnesal dehydrogenase from *P*. *minus* was analysed using MASCOT. However, MASCOT result did not reveal any identical tryptic peptides with significant scores. This could occur due to the uncharacterized genome of *P*. *minus* [64]. Insignificant score from MASCOT results suggested that the purified enzyme might be a novel farnesal dehydrogenase. Since *P*. *minus* genome sequences are not known, a homology based search was performed [65]. The search was applied to other known full-length sequences of aldehyde dehydrogenases including terpene aldehyde dehydrogenases. Present result shows that there were five peptides identified from the purified enzyme (Table 2). All 5 peptide sequences (A, B, C, D and E) showed similarity (50–100%) to several aromatic aldehyde dehydrogenases, short chain dehydrogenases and terpene aldehyde dehydrogenases, including *B*. *vietnamiensis G4* vanillin dehydrogenase, *P*. *putida p*-cumic aldehyde dehydrogenase, *N*. *domestica* short chain dehydrogenase, and *S*. *afghaniensis* geranial dehydrogenase. The homology comparison of the peptide sequence (A) with geranial dehydrogenase from *S*. *afghaniensis* showed a putative conserved domain for NAD-binding site as well as the catalytic residues (Gly255). Interestingly, two peptide sequences (A and B) showed similarity of 80% and 75%, respectively, with aldehyde dehydrogenase 3–1 from *A*. *aegypti* which reported able to oxidize farnesal [14]. This result suggested that the purified enzyme is likely a farnesal dehydrogenase.

**Table 2:** Identification of tryptic peptides from *P*. *minus* farnesal dehydrogenase.

Species	Peptide	E-value	Identity (%)
(A) The homology comparison of the peptide sequence from *P*. *minus* farnesal dehydrogenase showed a putative conserved domain for NAD-binding site as well as the catalytic residues.			
*P*. *minus*	RL**VT**AM**E**G**GSSKTAVNTG**RL	nd	nd
Geranial DH [*S*. *afghaniensis*]^1^	--**VT**-L**E**L**GG**K**S**-A**AV**	3.3	57
Aldehyde DH 3–1 [*A*. *aegypti*]^2^	-----------**KT**M**VN**	0.66	80
Aldehyde DH 1 [*A*. *annua*]^3^	--------**GSSK**S**A**	0.067	83
Short chain DH [*N*. *domestica*]^4^	-------------**AV**V**TG**	3.0	80
(B) The homology comparison of the peptide sequence from *P*. *minus* farnesal dehydrogenase showed no putative conserved domain.			
*P*. *minus*	RTLDLSGCT**GLSM**AS**LPRS**	nd	nd
Geranial DH [*S*. *afghaniensis*]^1^	---------------**LPRS**	0.11	100
Aldehyde DH 3–1 [*A*. *aegypti*]^2^	---------**GLSM**KF**LP**	0.044	75
(C) The homology comparison of the peptide sequence from *P*. *minus* farnesal dehydrogenase showed no putative conserved domain.			
*P*. *minus*	RAVSIM**KA**S**AVAF**ITNTASQRK	nd	nd
Geranial DH [*M*. *trichothecenolyticum*]^5^	------**KA**I**AVA**	0.54	83
Aldehyde DH 1 [*A*. *annua*]^3^	-**AV**---**KA**ARE**AF**	9.9	50
Vanillin DH [*B*. *vietnamiensis*]^6^	---------**AVAF**	0.2	100
(D) The homology comparison of the peptide sequence from *P*. *minus* farnesal dehydrogenase showed no putative conserved domain.			
*P*. *minus*	RVGFYNP**RAAEGEES**LRV	nd	nd
Geranial DH [*A*. *atlanticus*]^7^	---------**AE**D**EE**	0.21	80
Short chain DH [*P*. *citronellolis*]^8^	-------**RAAE**	0.082	100
Short chain DH [*N*. *domestica*]^4^	----------**EG**A**ES**	0.18	80
(E) The homology comparison of the peptide sequence from *P*. *minus* farnesal dehydrogenase showed no putative conserved domain.			
*P*. *minus*	IIAHADPSTVG**P**Q**L**I**LADLD**R	nd	nd
Geranial DH [*C*. *defragrans*]^9^	----------------**ADLD**	0.13	100
Vanillin DH [*B*. *vietnamiensis*]^6^	---------**P**V**L**V**L**DD**ADLD**	0.019	64
*p*-Cumic aldehyde DH [*P*. *putida*]^10^	----------------**ADLD**	0.14	100

*Streptomyces afghaniensis* (geranial dehydrogenase; WP_020275925.1)^1^, *A*. *aegypti* (yellow fever mosquito) (aldehyde dehydrogenase 3–1; AGI96738.1)^2^, *Artemisia annua* (sweet wormwood) (aldehyde dehydrogenase 1; ACR61719.1)^3^, *Nandina domestica* (heavenly bamboo) (short chain dehydrogenase; ACN87275.1)^4^, *Microbacterium trichothecenolyticum* (geranial dehydrogenase; KJL42303.1)^5^, *Burkholderia vietnamiensis G4* (vanillin dehydrogenase; ABO56825.1)^6^, *Altererythrobacter atlanticus* (geranial dehydrogenase; AKH43916.1)^7^, *Pseudomonas citronellolis* (probable short-chain dehydrogenase; ABC69246.1)^8^, *Castellaniella defragrans* (geranial dehydrogenase; WP_043683932.1)^9^, *Pseudomonas putida* (p-cumic aldehyde dehydrogenase; AAB62298.1)^10^

nd- Not determined.

### Effects of temperature and pH on enzyme activity

**Fig 5:**
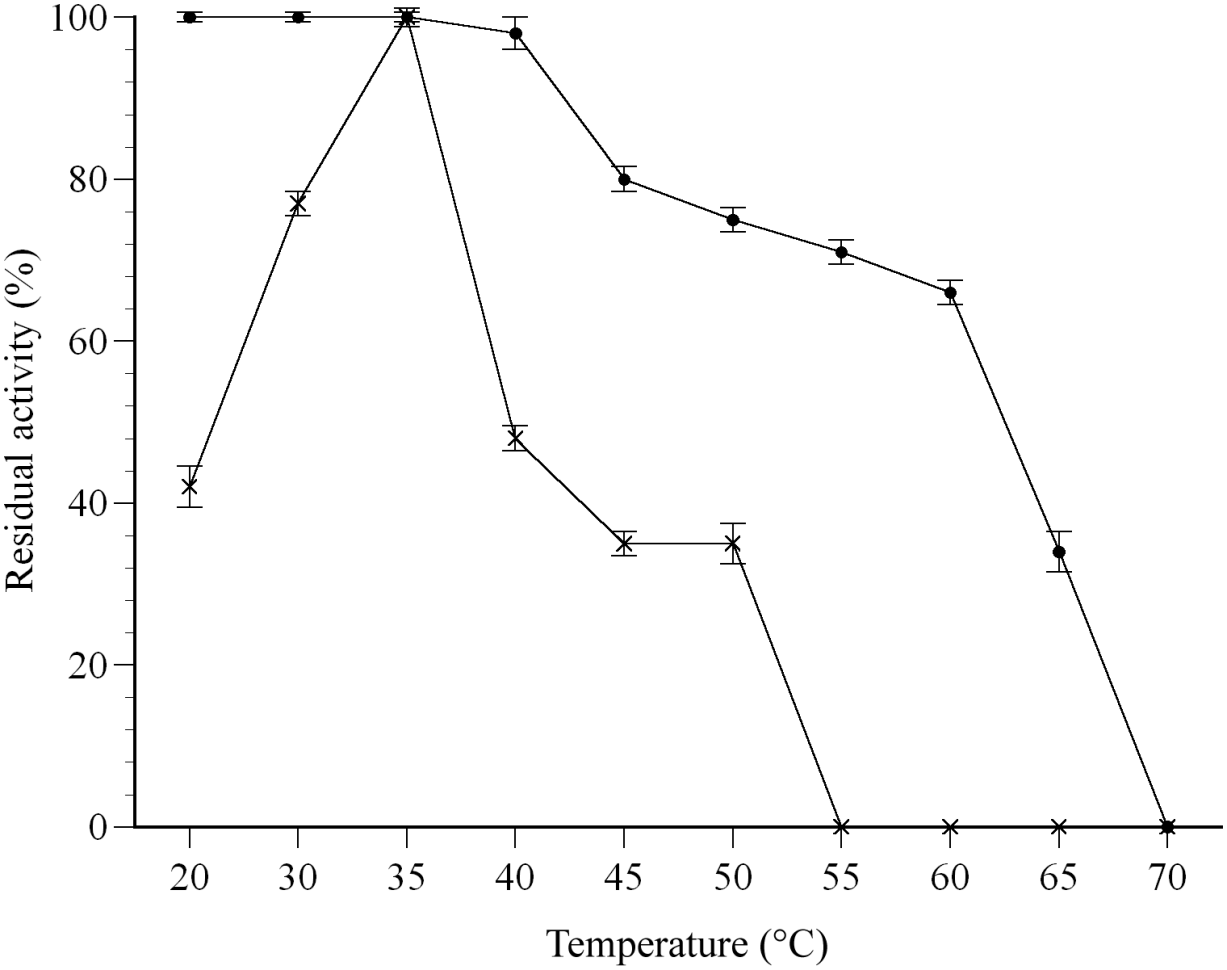
Effect of temperature towards activity of farnesal dehydrogenase. (**×**) Temperature optimum, (●) residual activity after heat treatment. The optimal temperature was determined by performing the standard enzyme assay as described in “Materials and Methods,” except that the reaction temperature was varied. The effect of temperature on residual activity of the enzyme was determined by incubating the purified enzymes at a temperature in the range of 20–70°C for 10 min at pH 7.5 (100 mM tricine-NaOH containing 2.5 mM 2-ME). Relative activity values for temperature are indicated as mean values (n = 3).

The effect of temperature was tested in the range 25°C to 70°C with farnesal as a substrate. The results show that the *P*. *minus* farnesal dehydrogenase has optimum temperature at 35°C (Fig 5). The result was comparable towards aldehyde dehydrogenases from *Hypericum androsaemum* [66] and *Escherichia coli* K-12 [47] where the optimum temperature of both enzymes were 35°C and 37°C, respectively. However, aldehyde dehydrogenases from *Spinacia oleracea*, *Avena sativa* and *Rattus norvegicus* [67–69] showed higher optimal temperatures at higher than 40°C.

The residual activity of the farnesal dehydrogenase after heat treatment was also examined (Fig 5). The enzyme retained more than 90% of its activity following treatment at 40°C for 10 min, suggesting the enzyme was highly active below 40°C. However, incubation at temperature above 60°C rapidly inactivated farnesal dehydrogenase activity and the enzyme was completely inactivated at 70°C. The results show a pattern where enzyme- catalysed reactions have rates that increase with temperature and becomes vulnerable to denaturation when the enzyme achieved its temperature limit [70].

**Fig 6:**
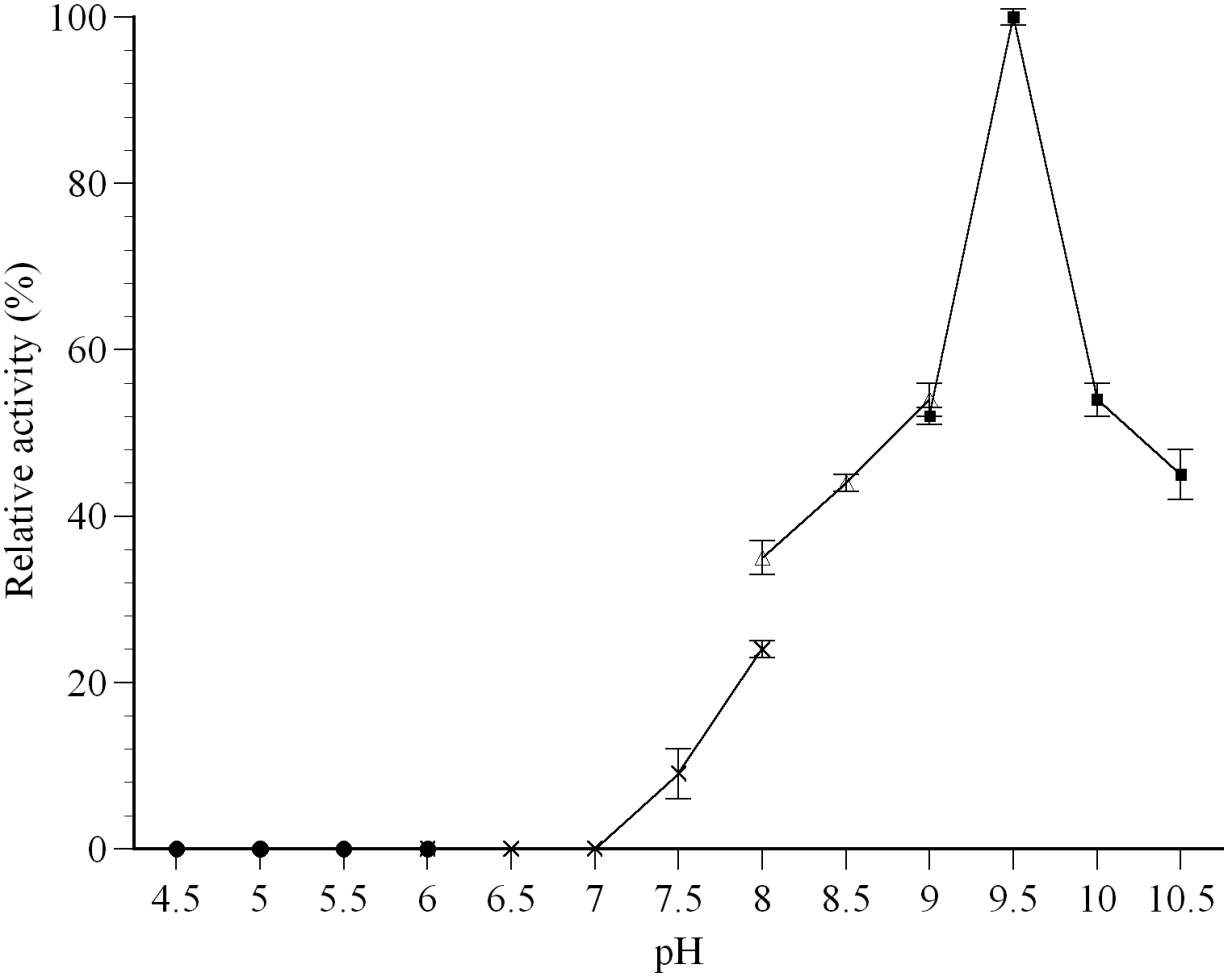
Effects of pH on farnesal dehydrogenase activity. Enzyme activity was assayed under the standard assay conditions, except that the following buffers were used at a final concentration of 100 mM in the incubation mixture: sodium citrate buffers (●), potassium phosphate buffers (**×**), tris-HCl buffers (Δ), and glycine-NaOH buffers (■). Relative activity values for pH are indicated as mean values (n = 3).

The optimal pH for farnesal dehydrogenase was estimated by monitoring the enzyme activity between pH 4.5–10.5 using various buffers at a concentration of 100 mM (Fig 6). The result shows the relative activity of farnesal dehydrogenase increased with the increment of pH from 7.0 and maximized at pH 9.5. *P*. *minus* farnesal dehydrogenase activity was highly sensitive to the environment pH where the enzyme remained active at pH values ranging from 9.0 to 10.0 and decreased remarkably when the pH value was lower than 8.0 or higher than 9.5. Increasing or decreasing one pH unit from the optimal pH (pH 9.5) attenuated about 50–70% of its activity. At pH 7.5, only 9% of the activity remained. The enzymatic activity was completely abolished at pH less than 6.5. These results suggest that the enzyme is active in an alkaline environment. The optimum pH of farnesal dehydrogenase is similar to other plant aldehyde dehydrogenases from *A*. *thaliana*, *I*. *batatas* and *Simmondsia chinensis* which have optimal activity at approximately pH 8, 8.8, and 9.0, respectively [52,53,71].

### Effects of inhibitors and metal ions towards farnesal dehydrogenase activity

The effects of inhibitors and metal ions on the enzyme activity were examined. The activity of the enzyme was measured after incubation of purified farnesal dehydrogenase with the tested compounds at the final concentration (0.1 mM) for 10 min at 35°C (Table 3). The inhibitor used as sulfhydryl agents were *p*-chloromercuribenzoate (pCMB), and iodoacetamide while the chelating agents used were sodium azide, and 1,10-phenantroline. Both sulfhydryl agents and chelating agents had a strong inhibitory effect (60–80% inhibition) towards the enzyme. Inhibition of the enzyme by sulfhydryl agents suggest that the enzyme has sulfhydryl group in its active site which is important for enzymatic activity. These results were consistent with the effects of sulfhydryl agent towards other aldehyde dehydrogenase activities that was found to greatly inhibited by pCMB [43,44,53,72,73] and iodoacetamide [43,45,53,73]. During chromatographic separations, *P*. *minus* farnesal dehydrogenase was observed to easily lost its activity in the absence of 2-ME indicates that this enzyme may likely a sulfhydryl-enzyme [74]. Inhibition by chelating agents suggests that metal component(s) may act as cofactor in the farnesal dehydrogenase activity [75]. Sodium azide has been described to inhibit both iron- and copper-containing enzymes [76] while 1,10-phenantroline interferes with the operation of an iron-containing enzyme only [77,78]. The enzyme activity was also strongly inhibited by metal ions Ag^+^, Cu^2+^ and Fe^3+^ (90–100% inhibition) while Ca^2+^, and Li^+^ inhibited the enzyme for less than 80% inhibition. These results suggest that Ag^+^ (heavy metals), Cu2^+^ and Fe3^+^ (transition metal) have a strong affinity towards free sulfhydryl groups which may be linked to the activity or for the native conformation of the enzyme. The metal ions may inhibit the enzyme by forming complexes with the sulfhydryl groups of cysteine side chains in the active site [46,79]. In support of the statement, structural analysis of aldehyde dehydrogenase from *Klebsiella pneumonia*, which also strongly inhibited by the metals was found to contain cysteine (Cys302) residue in the catalytic site [46]. Moreover, similar inhibitory effects of the metal ions used in this study were also previously described for other aldehyde dehydrogenases enzyme [43,45–48,53].

**Table 3:** Effect of metal ions and inhibitors on farnesal dehydrogenase activity with farnesal as a substrate and NAD^+^ as a coenzyme.

Inhibitor (0.1 mM)	Relative activity (%)
None	100±0.10
**Sulfhydryl agent**	
pCMB	21±2.00
Iodoacetamide	32±2.45
**Chelating agent**	
Sodium azide	20±1.72
1,10-Phenanthroline	15±1.00
**Metal ion**	
AgNO_3_	0
CuSO_4_	0
FeCl_3_	15±1.65
LiCl	26±1.40
CaCl_2_	27±2.00

Relative activity values are indicated as mean values (n = 3).

### Substrate specificity and kinetic parameter of farnesal dehydrogenase

*P*. *minus* farnesal dehydrogenase requires only NAD^+^ for the oxidation of farnesal while NADP^+^ was ineffective as a coenzyme (Table 4). The result suggests that *P*. *minus* farnesal dehydrogenase catalyzes the oxidation of aldehyde substrate in a NAD^+^-dependent manner. NAD^+^ has also been found as coenzyme for several other aldehyde dehydrogenases from plants such as *Cyperus iria*, *I*. *batatas*, *A*. *thaliana*, and *O*. *sativa* [28,52,53,80] and from insects such as *A*. *aegypti*, and *M*. *sexta* [14,20]. There is no reversible activity observed for farnesal dehydrogenase of *P*. *minus* when the enzyme was assayed with *trans- trans* farnesoic acid in the presence of NADH or NADPH.

**Table 4:** Studies of substrate specificity, coenzyme specificity and kinetic parameters of purified farnesal dehydrogenase from *P*. *minus*.

Substrate	Relative Activity (%)	*K*_m_ value (mM)	*V*_max_ (μmol/min)	*V*_max_/ *K*_m_
Farnesal	100±0.10	0.13	0.50	3.85
Citral	27±0.45	0.69	0.48	0.70
Citronellal	0	nd	nd	nd
*Trans*- cinnamaldehyde	33±0.59	0.86	0.33	0.38
α- Methylcinnamaldehyde	31±0.95	1.28	0.31	0.24
α- Bromocinnamaldehyde	0	nd	nd	nd
α- Amylcinnamaldehyde	0	nd	nd	nd
Decanal	0	nd	nd	nd
Octanal	0	nd	nd	nd
NAD^+^	100±0.10	0.31	0.48	1.55
NADP^+^	0	nd	nd	nd

Relative activity values are indicated as mean values (n = 3).

n.d- not determined.

The substrate specificity of farnesal dehydrogenase was examined using various allylic aldehydes, non-allylic aldehydes, aliphatic aldehydes and aromatic aldehydes (Fig 1).

The results (Table 4) show that *P*. *minus* farnesal dehydrogenase is highly specific for farnesal. Citral, *trans*- cinnamaldehyde and α- methyl cinnamaldehyde are poor substrates for the enzyme with relative activity of approximately 30% compared to farnesal. Other aromatic aldehydes tested were not oxidized by the enzyme. The enzyme also has no activity with non allylic aldehydes, cyclic aldehydes and aliphatic aldehydes tested in this study.

The result also shows the lowest *K*_m_ value for farnesal followed by citral, *trans*- cinnamaldehyde and α- methyl cinnamaldehyde (Table 4). The *V*_max_/*K*_m_ value which indicates the efficiency of the enzyme in catalyzing very low concentration of substrate was found decreased when other aldehydes were used as substrate in compared tofarnesal. This result indicates that the enzyme efficiently oxidized farnesal and therefore suggested that farnesal is the best substrate among all the tested aldehydes. Thus, it is proposed that the purified enzyme in this study is farnesal dehydrogenase that may able to oxidise farnesal in the later steps of JH III pathway.

Farnesal (3,7,11-trimethyl-2,6,10-dodecatrienal) with three double bonds positioned at C-2, C-6 and C-10 (Fig 1) was further compared with other substrate which oxidized by the enzyme. Lost of one double bond towards farnesal at C-10 will produce citral (3,7-dimethyl-2,6-octadienal) that has a *K*_m_ value of 5 times higher compared to farnesal. Citronellal (3,7-dimethyl-6-octenal), with double bond only at C-2 showed no activity when act as substrate towards *P*. *minus* farnesal dehydrogenase. Therefore, *P*. *minus* farnesal dehydrogenase was suggested to only oxidise substrate which has double bond at C-2 and C-6. The position of the double bond within the substrate is important to the enzyme as it may involve in both substrates binding and/or initiate a catalytic event [81].

In this study, a putative farnesal dehydrogenase of *P*. *minus* was found to oxidise aromatic aldehyde which contains an alkenyl group located between aromatic ring and carbaldehyde centre such as *trans*- cinnamaldehyde and *α*- methyl cinnamaldehyde. *Trans-* cinnamaldehyde was oxidised with *K*_m_ values of approximately 7 times higher than for farnesal, whereas *α*- methylcinnamaldehyde with the highest *K*_m_ values of approximately 10 times higher than for farnesal. Other tested aromatic aldehydes in the absence of the alkenyl group were unable to be oxidized by the enzyme. This result suggested that alkenyl group located between aromatic ring and carbaldehyde centre may help correctly position the substrate in the active site for reaction to occur [82].

Substitution of amyl and bromine group at α carbon of *α*- methylcinnamaldehyde yields *α*- amylcinnamaldehyde and *α*- bromocinnamaldehyde respectively, were not oxidised by the enzyme. These results suggested that cinnamaldehyde derivatives with the larger substituent group are affected by factors such as steric hindrance. Similar results have been obtained with aldehyde dehydrogenase 3A1 [83] and salivary aldehyde dehydrogenase [84] from *H*. *sapiens* where various sizes and type of substituents of the substrates tested affect the enzyme activity due to steric factors within the binding site.

In conclusion, the characteristic of putative *P*. *minus* farnesal dehydrogenase which is highly specific towards farnesal while oxidized aromatic aldehydes as substrates, whereas aliphatic aldehydes were not oxidized suggested that the enzyme was significantly different from the reported aldehyde dehydrogenase [1,14,17,20].

## Conclusion

In this report, we purified and characterized putative farnesal dehydrogenase which catalyzes the oxidation of farnesal to farnesoic acid from *P*. *minus* leaves. The purified enzyme exhibits similar physicochemical properties to other aldehyde dehydrogenases despite the distinctive substrate specificity. In contrast to other aldehyde dehydrogenases which have broad substrate specificity, *P*. *minus* farnesal dehydrogenase was highly specific to farnesal. Citral, cinnamyl aldehyde, and α- methyl cinnamaldehyde were poor substrates, whereas other aldehydes including aliphatic and aromatic aldehydes were not oxidized. Furthermore, two peptide sequences from MALDI-TOF/TOF-MS/MS analysis were found to share similarity with those previously reported aldehyde dehydrogenase that able to utilize farnesal as the substrate. We proposed that the enzyme purified and characterized in this study is a novel putative farnesal dehydrogenase from *P*. *minus*. It is likely that the function of this enzyme may be specifically to oxidize farnesal in the later steps of JH III biosynthesis pathway. It will be of interest therefore to determine its specific physiological role of farnesal dehydrogenasein JH III biosynthetic pathway in plant. This report therefore will provide valuable information for future recombinant protein production of the farnesal dehydrogenase. Complete sequence, structure and functional analysis of the enzyme were important for developing insect-resistant crop plants by deployment of transgenic plant.

## Supporting Information

S1 Fig Native polyacrylamide gel electrophoresis of protein from first SEC and second SEC. A: protein (7 μg) from first SEC fractions. The gel was stained with silver staining using PlusOne Silver Staining Kit, Protein (GE Healthcare, Uppsala, Germany); B: Purified farnesal dehydrogenase (7.3 μg) from second SEC fractions. Lane 1, silver staining; lane 2, activity staining. The reaction mixture contained 100 mM of glycine-NaOH buffer (pH 9.5), 2 mM of farnesal in ethyl acetate, 54 μM of 1-methoxy phenazine methosulphate, 0.3 mM of nitroblue tetrazolium, and 1 mM of NAD^+^. The protein was loaded upon native-PAGE using 15% of separating gels at pH 8.8 and 4% of stacking gels at pH 6.8, following the Laemmli buffer system without SDS [41]. The arrow indicates the position of protein band and activity of farnesal dehydrogenase detected on Native-PAGE.
(TIF)

S2 Fig Isoelectric focusing of purified farnesal dehydrogenase. The arrows indicate the protein bands approximately at pI 6.6. ReadyStrip IPG strips are preprinted to indicate anode end and pH range.
(TIF)
