# Supplementary figures and images for: Novel NAD+-Farnesal Dehydrogenase from Polygonum minus Leaves. Purification and Characterization of Enzyme in Juvenile Hormone III Biosynthetic Pathway in Plant
Source: PLoS One. 2016 Aug 25;11(8):e0161707. doi: 10.1371/journal.pone.0161707 (PMC4999093; doi:10.1371/journal.pone.0161707)

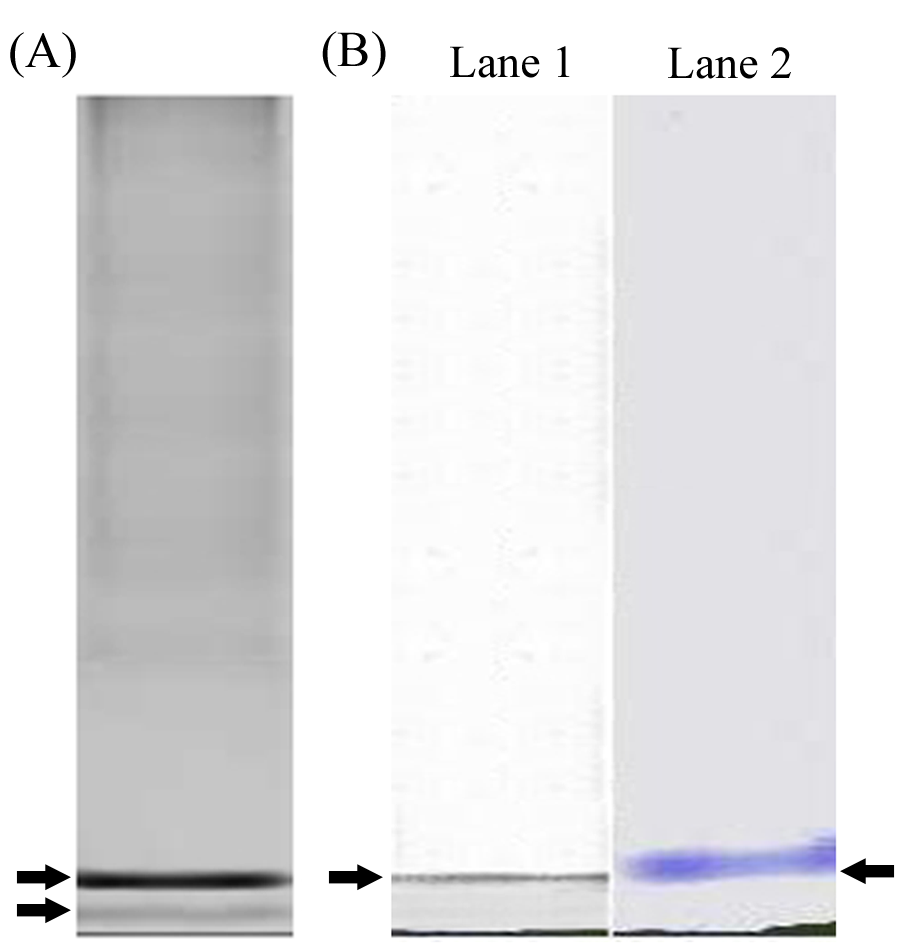

Supplement: S1 Fig — A: protein (7 μg) from first SEC fractions. The gel was stained with silver staining using PlusOne Silver Staining Kit, Protein (GE Healthcare, Uppsala, Germany); B: Purified farnesal dehydrogenase (7.3 μg) from second SEC fractions. Lane 1, silver staining; lane 2, activity staining. The reaction mixture contained 100 mM of glycine-NaOH buffer (pH 9.5), 2 mM of farnesal in ethyl acetate, 54 μM of 1-methoxy phenazine methosulphate, 0.3 mM of nitroblue tetrazolium, and 1 mM of NAD+. The protein was loaded upon native-PAGE using 15% of separating gels at pH 8.8 and 4% of stacking gels at pH 6.8, following the Laemmli buffer system without SDS [41]. The arrow indicates the position of protein band and activity of farnesal dehydrogenase detected on Native-PAGE. (TIF) [file pone.0161707.s001.tif]

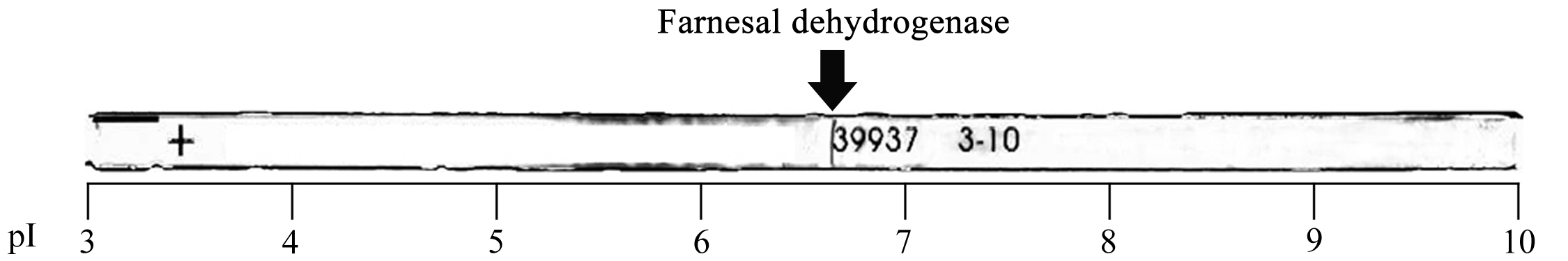

Supplement: S2 Fig — The arrows indicate the protein bands approximately at pI 6.6. ReadyStrip IPG strips are preprinted to indicate anode end and pH range. (TIF) [file pone.0161707.s002.tif]
